# High turnover drives prolonged persistence of influenza in managed pig herds

**DOI:** 10.1098/rsif.2016.0138

**Published:** 2016-06

**Authors:** Virginia E. Pitzer, Ricardo Aguas, Steven Riley, Willie L. A. Loeffen, James L. N. Wood, Bryan T. Grenfell

**Affiliations:** 1 Department of Epidemiology of Microbial Diseases, Yale School of Public Health, Yale University, New Haven, CT 06520, USA; 2 Fogarty International Center, National Institutes of Health, Bethesda, MD 20850, USA; 3 MRC Centre for Outbreak Analysis and Modelling, Department of Infectious Disease Epidemiology, School of Public Health, Imperial College, London SW7 2AZ, UK; 4 Department of Virology, Central Veterinary Institute, part of Wageningen UR, Lelystad 8200AB, The Netherlands; 5 Disease Dynamics Unit, Department of Veterinary Medicine, University of Cambridge, Cambridge CB3 0ES, UK; 6 Department of Ecology and Evolutionary Biology, Princeton University, Princeton, NJ 08544, USA

**Keywords:** influenza, swine, surveillance, mathematical modelling, transmission dynamics

## Abstract

Pigs have long been hypothesized to play a central role in the emergence of novel human influenza A virus (IAV) strains, by serving as mixing vessels for mammalian and avian variants. However, the key issue of viral persistence in swine populations at different scales is ill understood. We address this gap using epidemiological models calibrated against seroprevalence data from Dutch finishing pigs to estimate the ‘critical herd size’ (CHS) for IAV persistence. We then examine the viral phylogenetic evidence for persistence by comparing human and swine IAV. Models suggest a CHS of approximately 3000 pigs above which influenza was likely to persist, i.e. orders of magnitude lower than persistence thresholds for IAV and other acute viruses in humans. At national and regional scales, we found much stronger empirical signatures of prolonged persistence of IAV in swine compared with human populations. These striking levels of persistence in small populations are driven by the high recruitment rate of susceptible piglets, and have significant implications for management of swine and for overall patterns of genetic diversity of IAV.

## 1. Introduction

The H1N1 virus that caused the ‘swine influenza’ pandemic of 2009 raised concern over how little we know about the circulation and evolution of swine influenza A viruses (IAVs), despite the recognized importance of the pig for the evolution of human IAV. Current swine influenza surveillance programmes are usually dependent on opportunistic investigation of clinical incidents, with some notable exceptions [1–3]. However, the scale of such investigations is not obviously planned, nor is it clear at what grain virological surveillance of swine would be required in order to detect circulating variants capable of sustained transmission. A fundamental unknown is the population scale at which IAV usually persists. This constrains both the ability to plan surveillance and swine management programmes to limit regional or international IAV circulation.

There are a variety of empirical data, as well as some anecdotal evidence [4], that suggest swine influenza may be capable of persisting at small population scales, possibly even the individual farm level. Phylogenetic analyses suggested that precursors of the 2009 pandemic A/H1N1 virus, or at least its various gene segments, had been circulating undetected in swine populations for several years prior to its emergence [5,6]. Detecting individual IAV lineages would be more difficult if the scale of persistence were small. The global phylogeny and evolution of swine IAVs are recognized to be quite different to those of human IAV. For instance, the evolutionary dynamics of IAV in pigs do not appear to be associated with antigenic selection [1,7,8], while there is considerable geographical separation of swine IAV, both globally and within the USA [9,10]. We hypothesize, along with others [8,11,12], that the high, non-seasonal birth rate in pigs (approx. 20 piglets per sow per year) produces a steady supply of young susceptible pigs which promotes the persistence of IAV once it has been introduced into a herd.

The influence of population demographics on patterns of disease persistence is well established in human infectious disease epidemiology. Bartlett [13] was the first to analyse the likelihood of localized elimination, or fade-out, of measles as a function of community size and noted small populations experienced more frequent fade-outs than larger populations. He introduced the concept of a ‘critical community size’ (CCS) above which fade-out of infection was unlikely to occur. For measles, estimates of the CCS from developed country data have consistently placed it between 250 000 and 500 000 individuals, which can be reproduced by stochastic simulation models [14,15].

Influenza in humans is even more fragile. Evidence suggests human influenza disappears each summer in the high-latitude regions even from populations consisting of a billion people [16,17]. Whereas human influenza is characterized by strongly seasonal epidemics and frequent fade-out of infection during the summer months, swine influenza exhibits only weak seasonality in temperate regions, and IAV can be isolated year-round in countries such as the USA and UK [3,4]. However, even active surveillance for influenza on pig farms has been insufficient to fully understand how and why influenza is able to persist within swine populations [18].

Here, we use mathematical models titrated against seroprevalence data to estimate the ‘critical herd size’ (CHS) for influenza persistence in swine herds using a range of commonly found, realistic farm sizes and demographic parameters. Key questions addressed include: How large do the different farm types need to be for influenza to persist at the farm level? How is persistence affected by modifiable farm management strategies, such as the assumed farrowing interval, cohorting of pigs into age groups and the separation of weaners, growers, finishers and piglets and sows into different buildings or sites? How does the presence of pre-existing immunity in the population affect the probability of persistence? We then reassess the global phylogenetic evidence for comparative IAV persistence in humans and swine.

## 2. Material and methods

### 2.1. Model development

We developed a stochastic model to describe the transmission dynamics of IAV in pig herds under different management strategies, and examined how the probability of persistence varies with herd size and the basic reproductive number (*R*_0_, defined as the average number of infections caused by a single individual in a fully susceptible population). We used a basic *MSIR* (maternal immunity–susceptible–infectious–recovered) model in which pigs can fall into one of four possible infection states:

(i) *M*: protected by maternal antibodies and immune to clinical infection,
(ii) *S*: susceptible to infection,
(iii) *I*: infected and infectious to others and
(iv) *R*: recovered and immune.

Transitions between the different infection states occurred in a probabilistic manner according to a Poisson process. We updated the states at a sufficiently small time interval *τ* = 0.1 week such that the likelihood of two events occurring in one individual during the same time interval was low. The rates and transitions are defined in table 1.

**Table 1.** Parameters describing influenza transmission dynamics in swine.

event	transition	parameter	value	references
waning maternal immunity	*M* → *S*	*ω*_m_	1/6 week^−1^	[19]
infection of susceptible pigs	*S* → *I*	*R*_0_*γS*(*t*)	varies	
recovery from infection/infectiousness	*I* → *R*	*γ*	1 week^−1^	[20–22]
waning immunity from infection (in sows)	*R* → *S*	*ω_s_*	1/39 week^−1^	assumption

Estimates for the parameters needed to describe the transition probabilities in the model were obtained from the literature and expert consultations. The rate of waning of maternal antibodies was estimated from serological data collected during two longitudinal studies conducted in The Netherlands [19]. Following experimental infection, viral shedding has been demonstrated to commence as soon as 1 day post challenge and to last 3–10 days [20–22], yielding a mean estimate of the infectious period of 7 days. Models that assume conventional exponentially distributed infectious periods often overestimate the CCS [14]; thus, more realistic descriptions of the infectious period are a necessity when evaluating persistence. We divided the infectious state (*I*) into seven separate (daily) compartments such that the rate of recovery from infection is described by a gamma distribution, which more accurately captures variability in the infectious period. The resulting probability distribution for the infectious period was compared with data on viral shedding [20,22]. Since finishing pigs typically only live to 24 weeks of age, we conservatively assume that there is no waning of homologous immunity among previously infected finishing pigs during their lifetime. We explored a range of estimates for *R*_0_ and compared our model predictions for the proportions of pigs seropositive to the Dutch seroprevalence data [23].

We examined three common farm types:

(i) *farrow-to-finish farms*, ranging in size from 300 to 3000 growers/finishers (10–24 weeks old), as well as 180–1800 piglets/weaners (0–9 weeks of age) and 100–400 sows (up to 3 years of age).
(ii) *finishing farms* (*site III only*), ranging in size from 250 to 5000 growers/finishers (10–24 weeks old), but no piglets (0–3 weeks of age), weaners (4–9 weeks of age) or sows; and
(iii) *finishing farms* (*site II/III combined*), ranging in size from 250 to 5000 growers/finishers (10–24 weeks old) and 100–2000 weaners (4–9 weeks old), but no piglets or sows.

Ageing of pigs occurred deterministically, with the exception of gilts/sows on farrow-to-finish farms, which we assumed died or were replaced according to a Poisson process.

In the simplest case, we considered a finishing farm (site III only) in which new cohorts of 10-week old pigs were imported either each week or every three weeks (‘continuous flow’, as opposed to an ‘all-in-all-out’ system at the farm level). We assumed 22–24 week old pigs were sent to slaughter every three weeks, consistent with global industry practices. We assumed that growing pigs were sourced from farms of equivalent size per age group.

The rate of transmission-relevant mixing among pigs belonging to different age groups was described by ‘who acquires infection from whom’ (WAIFW) matrices. For finishing farms, we assumed there can be movement of infected pigs, but there is no indirect contact with pigs from other facilities, i.e. with piglets (0–3 weeks of age on average) for combined site II/III farms, or with piglets and weaners (0–10 weeks on average) for site III only farms. For pigs housed on the same farm, we explored a hierarchy of mixing assumptions, varying from homogeneous mixing among all pigs on the same farm to assortative age-specific compartment based mixing (see §2.3 for details).

To model influenza transmission on farrow-to-finish farms, we assumed that unweaned piglets (less than 3 weeks old) and sows were housed separately from weaners, growers and finishers, such that the mixing rate among piglets/sows, weaners/growers and finishers was 10 times higher than mixing between the different age groups. (See §2.3 for alternative mixing assumptions.) We explored the effect of farrowing occurrring every week or every three weeks; again, 22–24 week old finishers were sent to slaughter every three weeks. We assumed a population of 100–400 sows that were replaced at random intervals every 120 weeks on average, and examined a variety of assumptions about waning of immunity among sows; as a base case, we assumed immunity waned exponentially with a mean duration of 39 weeks.

We evaluated persistence under two different scenarios: (i) an *endemic population*, in which we assumed influenza had been circulating on the farm at a consistent level in the past and (ii) a *naive population*, in which we assumed that influenza was newly introduced such that there was no prior immunity among finishing pigs. For the endemic population scenario, we used a deterministic burn-in period of 3 years in order to obtain an equilibrium distribution of age-specific immunity in the population, which then determined our initial conditions. We then ran the stochastic model for 500 years, allowing for random reintroductions of infection at a mean rate of two reintroductions per year (with two infectious individuals per introduction). The probability of fade-out was defined as the proportion of years in which there were no infectious individuals on the farm for two or more consecutive weeks. The rate of reintroduction and definition of a fade-out are balanced in such a way that there is sufficient opportunity for reintroduction following a fade-out, but reintroductions are not so frequent that they could be mistaken for ongoing transmission [24–26]. We also examined the sensitivity of our conclusions to the frequency of reintroduction by varying the rate between one and 10 reintroductions per year. For the naive population scenario, we initialized the population with one infectious individual in each age group and no recovered/immune individuals. We ran the simulation 500 times for a duration of 1 year (reinitializing the model each time) and defined the probability of fade-out as the number of simulations in which there were no infectious individuals present at the end of the year. We defined the CHS for a given *R*_0_ as the minimum population size for which the probability of stochastic fade-out per year, *F*, was less than 0.05.

### 2.2. Model titration against serological data

Model predictions for the relationship between herd size and the probability of persistence were ground-truthed by comparing model output for the seroprevalence among finishing pigs at the end of the finishing period (22–24 weeks of age) to data on the seroprevalence of H1N1, H3N2 and H1N2 among finishers from three high­-density pig farming regions in The Netherlands [23] (see the electronic supplementary material, Dataset). The study included 15 Dutch finishing herds ranging in size from 290 to 4500 finishing pigs and 14 farrow-to-finish herds ranging in size from 450 to 1100 finishing pigs (70–430 sows) [23]. Vaccination of pigs against IAV was not allowed among farms participating in the study, so seropositivity was indicative of past infection. For further details on the data collection, see [23].

We placed constraints on the possible values of *R*_0_ by comparing model output for the cumulative proportion of pigs infected prior to slaughter (i.e. the proportion of the population in the recovered and immune *R* class at 22–24 weeks of age) or with lingering maternal antibodies (i.e. in the *M* class at 22–24 weeks of age, which on average was less than 0.5%) to the seroprevalence data described above. We calculated model output for both the mean seroprevalence at slaughter over the full duration of all simulations and 95% prediction intervals for the seroprevalence at slaughter based on the sampling time.

To examine whether there was a relationship between seroprevalence and finishing herd size among the Dutch herds, we used logistic regression to model the proportion of pigs seropositive versus the herd size, and examined whether the coefficient for the herd size was significantly different from zero. We conducted both univariate analyses and multivariate analyses, in which we also took into account the average compartment size for finishing pigs as well as the number of sows and piglets per compartment on farrow-to-finish farms.

In comparing to the Dutch seroprevalence data, we assumed seroconversion to H1N1, H1N2 and H3N2 among sampled pigs represented independent samples from farms of the same size [23]. Seropositivity to more than one subtype could be positively correlated (due to cross-reactivity among subtypes or common risk factors for infection) or negatively correlated (due to cross-immunity among subtypes). We also examined whether the relationship between seroprevalence and farm size held for the subtype-specific data, in addition to the data for all subtypes.

### 2.3. Sensitivity analyses

For each farm type, we examined the sensitivity of our model predictions to the frequency of introduction of weaned piglets (combined site II/III finishing farms), growers (site III finishing farms) or farrowing (farrow-to-finish farms). We ran the model assuming births/introductions occurred on a weekly basis (*continuous births*) or every three weeks (*batch farrowing*). For finishing farms, we also examined sensitivity to the introductions interval by assuming births/introductions occurred every six weeks or every 12 weeks. The extreme would be an ‘all-in all-out’ site management strategy, in which weaned piglets are only reintroduced once the previous cohort of finishing pigs is taken to slaughter, in which case we would need to allow for environmental persistence and/or reintroduction of IAV with each new cohort of piglets. Finally, we examined whether allowing for infection of piglets with maternal antibodies at a reduced rate (10-fold lower [27]) would affect our results.

We also examined the sensitivity of our model predictions to a hierarchy of mixing assumptions. The simplest assumption is that mixing is homogeneous at the farm level, i.e. all pigs on a given farm have an equal probability of being infected by an infectious pig regardless of age (*homogeneous mixing*). Alternatively, we assumed that pigs housed in the same accommodations (e.g. grower versus finisher accommodations) mix with one another at a rate that is 10 times greater than with those housed in different accommodations (*site-specific mixing*). Finally, we assumed pigs in the same age-specific compartment mix with one another at a rate that is 10 times higher than with those in separate compartments (*age compartment mixing*). We assumed compartments contain pigs in age groups 0–3 weeks, 4–6 weeks, 7–9 weeks, … , 19–21 weeks and 22–24 weeks of age, and explored model predictions assuming one to five separate compartments per age group. To estimate the farm-level *R*_0_ when mixing occurred within age compartments, we calculated the maximum eigenvalue of the next-generation matrix [28,29].

### 2.4. Phylogenetic analysis of global influenza A virus evolution in humans and swine

There are very few data on how the prevalence of influenza differs for farms of varying size. This prohibits a direct comparison between our model predictions and observed data on the persistence of influenza in swine herds of different sizes, such as Bartlett's seminal study of measles persistence [13].

Therefore, in an attempt to validate our model, we undertook a direct comparison of the global phylogenies of human and swine IAV to examine the evidence in favour of greater persistence of swine IAV lineages. We hypothesized that the ability of IAV to persist in very small populations would make samples from the same geographical region in different years more similar on average than is the case for human IAV. Essentially, we hypothesized that swine IAV lineages would exhibit longer branch lengths than IAV lineages isolated from humans.

Comparing swine and human evolutionary patterns is particularly challenging for IAV given the spatio-temporal sampling inconsistencies and occurrence of human-to-swine transmission events every few years. To circumvent these limitations, we devised a *post hoc* sequence sampling scheme that limits the number of sequences collected in each species (in both space and time) to overcome sampling biases, and purges putative lineage transposal sequences (viruses belonging to one species' genetic lineage which were collected in the other species). We chose to include only sequences from the USA, Europe and China, given their global geographical dispersion, relatively similar spatial scale and sequence availability. By sub-setting the sequences into regional groups according to USDA farm production region [9] or country, we can thus assess coherent space–time diversity in both the human and swine influenza lineages.

We applied the following sampling scheme to the set of human and swine H3 haemagglutinin (HA) sequences with more than 75% gene coverage available in GenBank. To avoid over-representation of human samples, we randomly sampled human to swine IAV at a ratio of one-to-one, matching the sequences based on region (USDA farm production region or country) and year of isolation. More specifically, for each spatial group and year, we picked an equal number of swine and human sequences at random, limiting our analysis to six samples per region per year to avoid temporal sampling biases. Phylogenetic analysis of the resulting set of sequences highlighted putative cross-species transmission events. Viruses more closely related (either in the same clade of a maximum-likelihood tree or simply having a closer genetic distance in terms of number of mutations) to viruses collected in a different host were removed from the analysis.

We then analysed the phylogeny of the final set of 382 HA sequences for each host separately using the maximum-likelihood method based on the JTT matrix-based model [30]. All positions containing gaps and missing data were eliminated. Evolutionary analyses were conducted in MEGA v. 6 [31].

To statistically describe differences between the human and swine trees, we calculated the average between region/country distances for both swine and human sequences. These distances summarize the number of amino acid substitutions per site from averaging over all sequence pairs between groups (regions). A two-sample Kolmogorov–Smirnov test comparing the distributions of these mean group distances was used to highlight significant differences in evolutionary pathways of human and swine viruses.

## 3. Results

### 3.1. Estimating the ‘critical herd size’ using mathematical models

**Figure 1.**
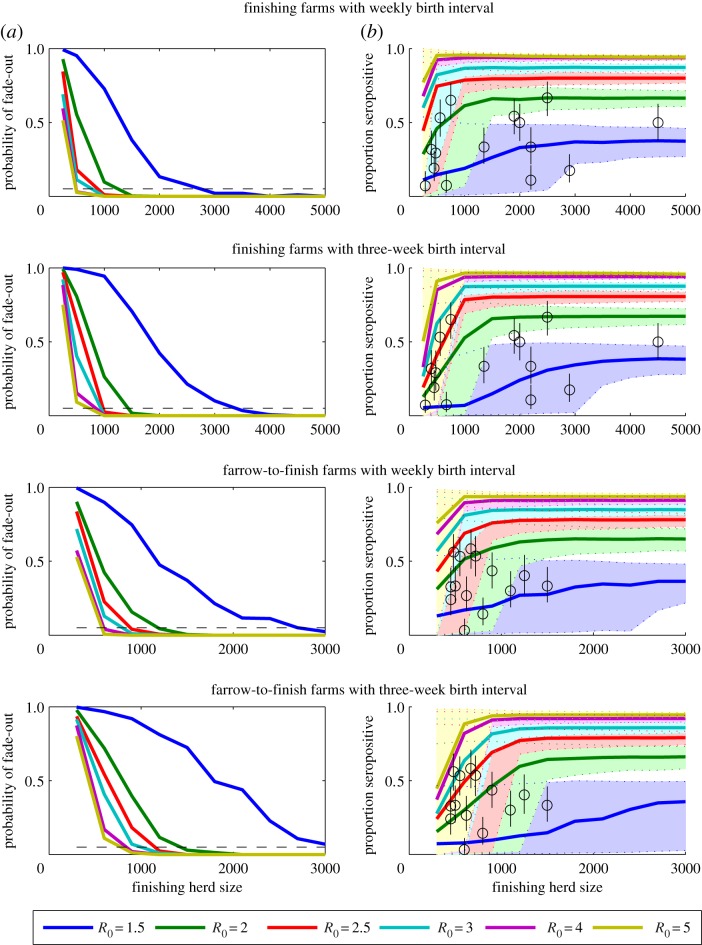
Model-predicted persistence and seroprevalence patterns of swine IAV on finishing and farrow-to-finish farms. (*a*) The probability of stochastic fade-out of infection is plotted for finishing herds ranging in size from 250 to 5000 pigs and farrow-to-finish farms with 300–3000 finishing pigs. The coloured lines represent the model-predicted probability of fade-out for values of *R*_0_ between 1.5 and 5, while the dashed black line represents *F* = 0.05. (*b*) The model-predicted mean seroprevalence of influenza prior to slaughter (22–24 weeks of age) is represented by the coloured lines, while the shaded regions between the dotted coloured lines represent the corresponding 95% prediction intervals. The black circles represent the mean seroprevalence of H1N1, H1N2 and H3N2 antibodies observed among finishing pigs from farms of varying size in The Netherlands, while the black lines are the corresponding 95% CIs. Births and the movement of pigs from one site to the next are assumed to occur weekly or every three weeks, as indicated.

**Figure 2.**
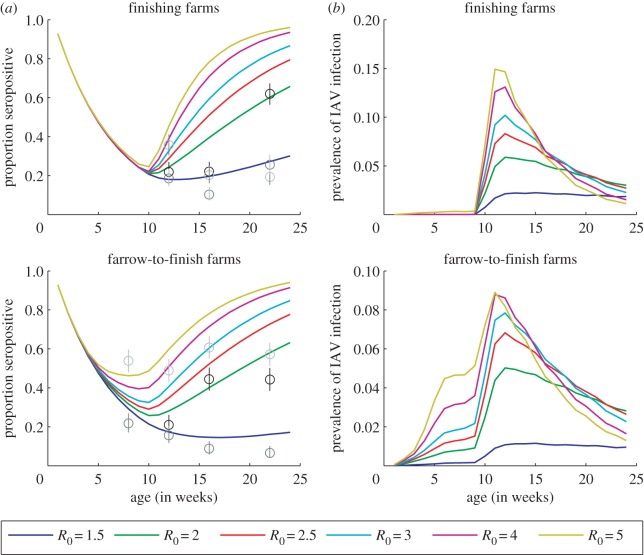
Seroprevalence and prevalence of IAV infection by age among pigs on finishing and farrow-to-finish farms. (*a*) The model-predicted mean seroprevalence of influenza by age (1–24 weeks of age) is plotted (coloured lines) for finish farms and farrow-to-finish farms with 1500 finishing pigs for values of *R*_0_ from 1.5 to 5. The circles represent the mean seroprevalence of H1N1 (black), H1N2 (light grey) and H3N2 (dark grey) antibodies observed among finishing pigs in The Netherlands (for farms of all sizes), while the lines are the corresponding 95% CIs. (*b*) The model-predicted mean prevalence of IAV is plotted by age (1–24 weeks of age) for finishing and farrow-to-finish farms. Births and the movement of pigs from one site to the next are assumed to occur every three weeks.

Based on our stochastic simulation model, we found that IAV was likely to persist in finishing herds with 1500 pigs when *R*_0_ ≥ 2 and in herds with at least 3000 pigs when *R*_0_ = 1.5 (figure 1*a*). Model output was generally consistent with seroprevalence data when *R*_0_ was 1.5–2.5, with the higher *R*_0_ values typically associated with the smaller farms (figure 1*b*). Fade-out of infection was slightly more likely to occur when new cohorts of pigs were imported every three weeks compared to every week, but overall the results were similar (figure 1). Results were fairly insensitive to the rate of reintroduction of influenza, particularly when persistence was common (*R*_0_ ≥ 2) (electronic supplementary material, figure S1). If we assumed there is no prior immunity in the population, persistence (for at least 1 year) was slightly more likely to occur, and variability in seroprevalence at slaughter was greater (electronic supplementary material, figure S2). The prevalence of IAV infection was predicted to peak early in the finishing period (approx. 12 weeks of age), but was generally less than 8% for values of *R*_0_ between 1.5 and 2.5 (figure 2). Most infections occurred after arriving at the finishing farm when finishing pigs were sourced from nurseries (site II) of equivalent group size (figure 2). Allowing for infection of piglets at a reduced rate increased the probability of persistence (electronic supplementary material, figure S3).

Infection was slightly less persistent on ‘farrow-to-finish’ farms compared to ‘finishing’ farms for a given value of *R*_0_ among finishing pigs (figure 1). However, the seroprevalence data were suggestive of slightly higher values of *R*_0_ among finishing pigs on farrow-to-finish farms compared to finishing farms. This is consistent with the younger age of peak seroprevalence observed previously (figure 2) [23]. For *R*_0_ = 2–4, the CHS for persistence on farrow-to-finish farms was estimated to be between 1200 and 2700 pigs when farrowing occurred every three weeks (figure 1). Again, the CHS was slightly lower when we assumed weekly farrowing. The number and waning of immunity among sows had only a slight impact on the CHS for farrow-to-finish farms (electronic supplementary material, figure S4).

Combined site II/III finishing farms are not common in The Netherlands, but are found in the UK and other parts of the world. In this case, we found persistence was greater (i.e. the CHS decreased) for the same finishing herd size compared with site III only finishing farms (electronic supplementary material, figure S5); however, the total number of pigs on the farm would be greater (by 40%) when accounting for 4–9 week old weaners.

**Figure 3.**
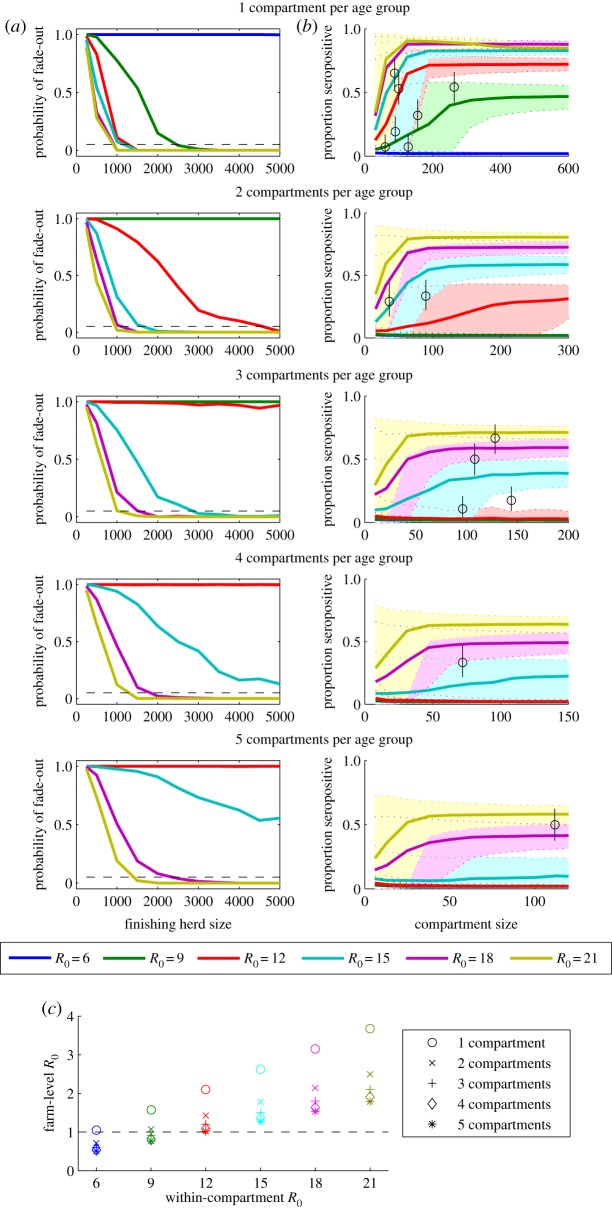
Impact of mixing assumptions on model-predicted persistence and seroprevalence patterns of swine influenza in finishing herds. (*a*) The probability of stochastic fade-out of infection is plotted for finishing herds ranging in size from 250 to 5000 pigs. We model 1–5 compartments per age group, and assumed the transmission rate among pigs within the same age-specific compartment is 10-times higher than the transmission rate among pigs in different compartments. The coloured lines represent the model-predicted probability of fade-out for values of *R*_0_ between 1.5 and 5, while the dashed black line represents *F* = 0.05. (*b*) The model-predicted mean seroprevalence of influenza prior to slaughter (22–24 weeks of age) is represented by the coloured lines, while the shaded regions between the dotted coloured lines represent the corresponding 95% prediction intervals. The black circles represent the mean seroprevalence of H1N1, H1N2 and H3N2 antibodies observed among finishing pigs from farms of varying size in The Netherlands, while the black lines are the corresponding 95% CIs. Total finishing herd sizes are indicated on the left-hand plots, while the corresponding compartment sizes for a given herd size and number of compartments are indicated on the right-hand plots. Births and the movement of pigs from one site to the next are assumed to occur every three weeks. (*c*) The relationship between the within-compartment *R*_0_ and the farm-level *R*_0_ depends on the number of compartments per age group.

Finishing farms tend to be highly structured, with pigs of a similar age and weight grouped together. Such mixing may facilitate influenza transmission among pigs sharing the same compartment. Direct transmission through pig-to-pig contact is thought to be the dominant mode of IAV transmission, but airborne and indirect transmission via fomites also occurs, albeit at slightly reduced rates [11,32,33]. If we assumed that the transmission rate is 10 times higher among pigs in the same compartment than between pigs housed in different compartments (organized by three-week age groups), we found the CHS for influenza persistence was considerably greater than when assuming homogeneous mixing for a given within-compartment *R*_0_ value; the CHS increased with the number of compartments per age group (figure 3). However, the seroprevalence data were most consistent with within-compartment *R*_0_ ≥ 9, again resulting in a CHS < 4000 pigs (figure 3). Experimental studies have estimated the *R*_0_ for influenza in closely mixed unvaccinated swine to be approximately 11 [22]. Our comparison with the seroprevalence observed under field conditions suggest that while this may be an appropriate reflection of transmission within age-specific compartments (reflecting the experimental design), the farm-level *R*_0_ is more likely on the order of 1.5–3 (figure 3), which is more consistent with values of the reproductive number estimated for pigs vaccinated with a heterologous strain [22]. Higher values of *R*_0_ would only lead to greater persistence and a lower CHS according to our analysis (for *R*_0_ ≤ 15; results not shown).

**Figure 4.**
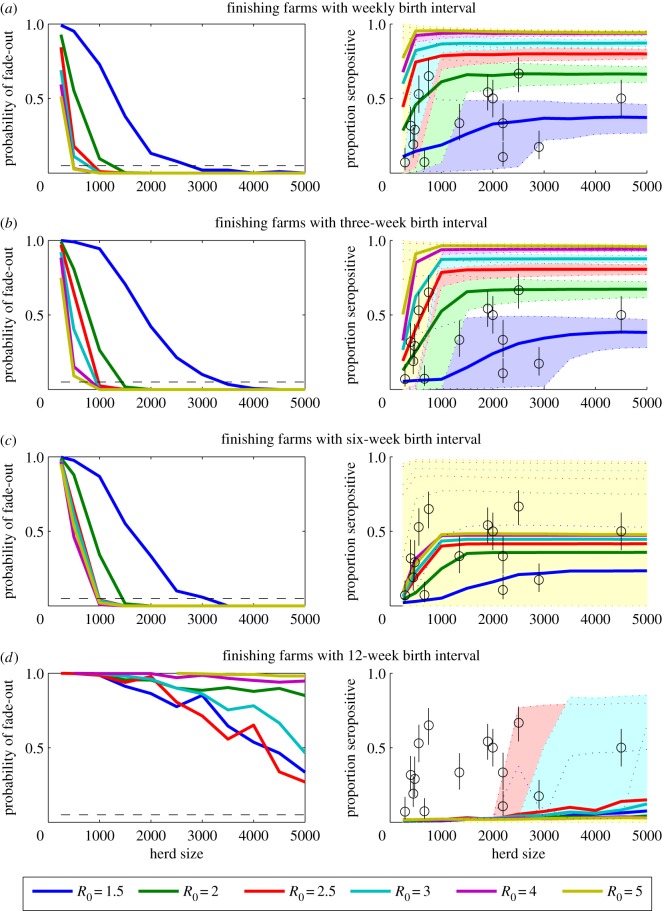
Sensitivity of results for model-predicted persistence to the frequency of introduction of weaners on finishing farms. The probability of stochastic fade-out of infection (left) and the model-predicted mean seroprevalence of influenza prior to slaughter (right) is plotted for finishing herds ranging in size from 250 to 5000 pigs. Births and the movement of pigs from one site to the next are assumed to occur (*a*) weekly, (*b*) every three weeks, (*c*) every six weeks or (*d*) every 12 weeks. The coloured lines represent the model results for values of *R*_0_ between 1.5 and 4, while the shaded regions between the dotted coloured lines represent the corresponding 95% prediction intervals. The black circles represent the mean seroprevalence of H1N1, H1N2 and H3N2 antibodies observed among finishing pigs from farms of varying size in The Netherlands, while the black lines are the corresponding 95% CIs.

As we increased the farrowing interval in our model from weekly births/introductions to introductions occurring every 12 weeks, the probability of persistence for a given value of *R*_0_ decreased, while the model-predicted variance in seroprevalence increased (figure 4). This suggests that it is the continuous reintroduction of new susceptible pigs and mixing among pigs of different ages that drives persistence. Therefore, a logical conclusion is that all-in-all-out management strategy would be less likely to support IAV persistence, provided there is no environmental transmission.

### 3.2. Relationship between finishing herd size and seroprevalence in the Dutch data

**Figure 5.**
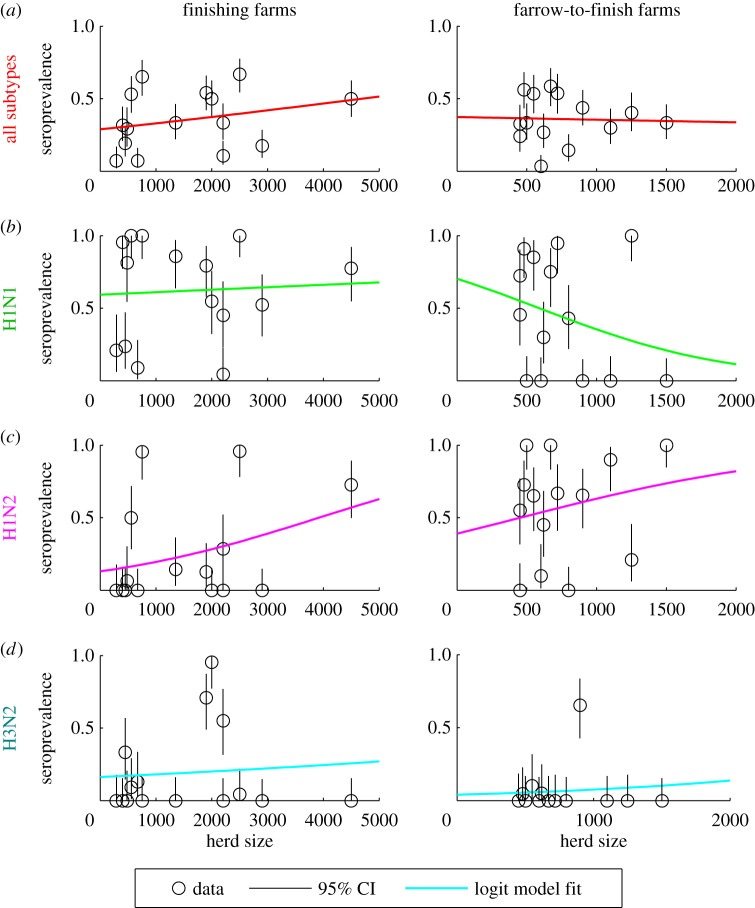
Relationship between finishing herd size and swine influenza seroprevalence among Dutch finishing and farrow-to-finish herds. The seroprevalence data (black circles) for (*a*) all subtypes, (*b*) H1N1, (*c*) H1N2 and (*d*) H3N2 are plotted against herd size for finishing farms (left) and farrow-to-finish farms (right). The black lines denote 95% binomial confidence intervals. The coloured lines show the best-fit univariate logistic regression model between herd size and mean seroprevalence. The coefficient for the relationship between seroprevalence and finishing herd size was significant for all subtypes on finishing farms (*β* = 1.9 × 10^−4^, *p* < 0.001), H1N1 on farrow-to-finish farms (*β* = −1.5 × 10^−3^, *p* < 0.001) and H1N2 on both finishing farms (*β* = 4.8 × 10^−4^, *p* < 0.001) and farrow-to-finish farms (*β* = 1.0 × 10^−3^, *p* < 0.05). All other coefficients for the relationship between seroprevalence and herd size were not significant (*p* > 0.05). (Online version in colour.)

We detected a weak but significant relationship between herd size and mean seroprevalence among finishing herds (*β* = 1.9 × 10^−4^, *p* < 0.001), but not farrow-to-finish herds (*β* = −8.0 × 10^−5^, *p* > 0.05); however, there was considerable farm-to-farm heterogeneity (figure 5). The relationship between finishing herd size and seroprevalence was strongest for the H1N2 subtype, which was the most common subtype among both finishing and farrow-to-finish farms (see the electronic supplementary material, Data). Weaker and/or negative relationships were observed for the other subtypes, possibly due to cross-immunity among subtypes (figure 5). In multivariate analyses, seroprevalence on finishing farms was positively associated with both the total number of finishing pigs and the average number of pigs per compartment (*p* < 0.01), while seroprevalence on farrow-to-finish farms was significantly (and positively) associated only with the number of sows (*p* < 0.05).

### 3.3. Phylogenetic analysis

**Figure 6.**
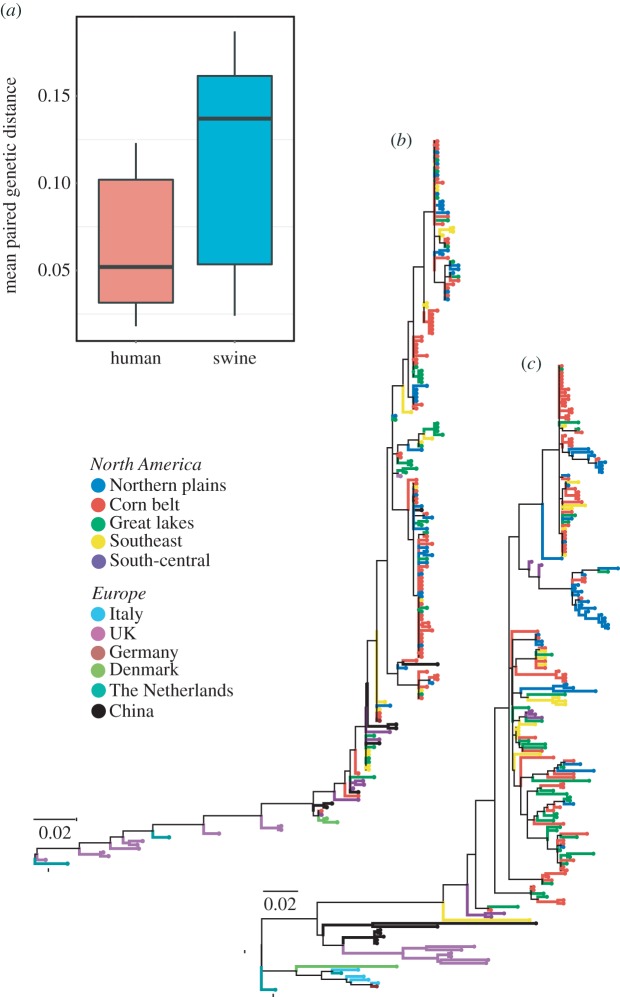
Phylogenetic analysis of H3 influenza virus sequences from human and swine. (*a*) Box plot of the average between region/country distances for both swine and human sequences. These distances summarize the number of amino acid substitutions per site from averaging over all sequence pairs between groups (regions). A two-sample Kolmogorov–Smirnov test was used to compare the distributions of these mean group distances. Phylogenetic trees with the highest resulting likelihood are displayed for (*b*) human IAV and (*c*) swine IAV. Branches are colour-coded according to region. The percentage of trees in which the associated taxa clustered together is shown next to the branches whose length scales with the number of substitutions per site.

A side-by-side comparison of the evolutionary trajectories of human and swine IAV reveals that swine virus lineages exhibit longer branch lengths (*p* < 0.0001; figure 6). Swine IAV also cluster more geographically than temporally compared with human influenza strains (figure 6). While these patterns are likely to be driven in part by interspecies differences in international movement and demography, the latter leading to greater immune pressures on human versus swine IAV [34], they are also suggestive of greater persistence of swine IAV.

## 4. Discussion

Both models and phylogenetic data point to remarkably strong local persistence of swine IAV. Our model suggests influenza viruses persist in swine populations more than 100-fold smaller than classical results for measles in humans, and 100 000-fold smaller than seasonal human influenza at high latitudes. Multidisciplinary approaches are needed to understand how the considerable differences between human and livestock demography influence the different patterns of persistence in zoonotic diseases. Essentially, high population turnover and constant influx of new susceptible pigs make continuous flow swine operations akin to ‘chemostats’ in which the growth medium is constantly replenished, facilitating local, long-term persistence of an otherwise epidemiologically fragile virus.

We have interpreted the differences in the phylogenetic patterns of IAV in humans and swine as evidence that IAV is able to persist in far smaller swine populations than human populations. Although based on maximum-likelihood trees, our approach is similar to recent work comparing different human influenza subtypes [35], in which it was found that influenza A/H1N1 and B are able to persist in smaller human populations than can influenza A/H3N2. Also, given that a signature of local persistence is visible in the sparse public data used here, our phylogenetic analyses clearly motivate longitudinal sampling of clustered farms in a few key regions. Such high-resolution phylogeographic data could rapidly confirm our main finding.

Only one other model (that we are aware of) has been developed to examine the transmission dynamics of influenza within swine herds [12,36]. Reynolds *et al.* [12] used a deterministic model to examine the dynamics of influenza and effectiveness of vaccination on two types of swine farms in the USA. While their analysis suggested that influenza is likely to persist on breeding farms (containing piglets, sows and gilts) with at least 250 sows and gilts, they only examined an all-in all-out management strategy for finishing farms [12], and deterministic models are not suitable to examine questions of persistence in such small populations. Mathematical modelling has also been used to examine the persistence of other pathogens in swine herds, including porcine reproductive and respiratory syndrome virus (PRRSV) [37,38] and *Salmonella typhimurium* [39]. These studies found that the probability of persistence increased with herd size, but the models used were specifically parametrized to address these other pathogens, and only examined a limited range of farm types, herd sizes, management strategies and mixing assumptions.

It has been difficult to assess the question of IAV persistence in swine populations through active surveillance. If influenza strains are indeed capable of persisting at the individual farm level, our model suggests that this may be very difficult to detect through conventional surveillance efforts, since prevalence on the farm is predicted to be low (less than 8%) when transmission is endemic (figure 2). One recent study from the USA found IAV could be detected on 29 of 32 farms over 12–24 months of surveillance and 21.7% of groups sampled monthly using nasal swabs [18]. However, in most cases only one pig tested positive, and the overall prevalence of IAV was 4.6% [18]. Similarly low rates of viral isolation were observed in abattoirs in the USA (2.2%) and Hong Kong (1.6%) [2,40]. Indeed, our model predicts that when persistence is occurring, the mean prevalence of IAV in finishing pigs is less than 6%, peaking at the beginning of the finishing period (figure 2). This suggests that more than 45 pigs would need to be sampled in a random sampling scheme to have a 95% probability of detecting at least 1 positive pig *at each time point for each strain*. This is an important consideration when sampling longitudinally compared to investigating disease outbreaks. Enhanced surveillance methods that permit sampling of a large number of pigs in order to detect influenza when the prevalence is low have only recently become more commonplace [41].

Detecting different strains at different time points on the same farm does not necessarily mean the persistence of one strain is not occurring or cannot occur. In addition to the sampling issues raised above, it is possible that when a new strain of IAV is introduced to a farm, it will have a fitness advantage over the existing strain due to the lower levels of immunity to the new strain. This could lead to strain replacement. Thus, greater connectivity among farms might be expected to lead to less overall diversity of IAV. A limitation of our modelling approach is that we only consider the dynamics of a single influenza strain. Expanding the model to include multiple strains is complicated and involves making uninformed assumptions about cross-immunity, and therefore is beyond the scope of the current study.

Our findings have important implications for both understanding the factors that led to the emergence of the 2009 H1N1 ‘swine flu’ pandemic as well as for evaluating how herd management strategies affect the persistence of other economically important livestock infections. Recent studies have found high levels of transmission of human IAV to swine [42]. Reassortment can occur when a host is simultaneously infected with two or more influenza strains, and is an important processes in the emergence of novel influenza strains [43]. Extensive reassortment has been observed among swine and human IAV circulating among pigs in the North America [9,44], Europe [45] and Asia [1]. These reassortant viruses can circulate undetected for years [1,5,6,46].

Efforts to curb the emergence of novel IAV in swine populations should focus on interrupting transmission at the farm level, particularly for finishing herds of more than 3000 pigs. Our analysis suggests increasing the interval between farrowing or the introduction of growers/finishers, such as an all-in-all-out management strategy, would be one way to decrease the persistence of novel strains (figure 5). Vaccination could also help to interrupt transmission by lowering the effective reproductive number, although this would need to be balanced against the selective pressures imposed by vaccines.

Identifying precursors to novel human-transmissible influenza strains circulating in swine before they make the jump to humans may be akin to the proverbial search for a needle in a haystack. In the USA alone, there are approximately 8300 swine operations consisting of at least 2000 pigs, and these operations account for more than 85% of the total inventory [47]. Intensive farming leading to the concentration of more pigs on fewer farms has become commonplace throughout the world [48]. Each large-scale swine operation could potentially harbour its own unique influenza strains, which could persist for decades. While surveillance plays an essential role in our understanding of the ecology and evolution of IAV in swine, it will be difficult to detect all circulating strains with pandemic potential.

Improvements in biosecurity and surveillance practice have been cited as important priorities by national and international regulatory bodies [49,50] and researchers worldwide [51]. Given the propensity for interspecies transmission of IAV between pigs and humans, more attention should be paid to the dynamics of influenza in swine populations, and to the workers who have contact with pigs and can serve as a bridge between the human and swine IAV populations. Understanding and identifying factors that promote the persistence of IAV among swine herds can help to inform strategies to eliminate the pathogen and decrease the risk of zoonotic transmission to humans.

## Supplementary Material

Figure S1. Sensitivity of model predictions to the rate of reintroduction of influenza on swine farms.

## Supplementary Material

Figure S2. Model-predicted persistence and seroprevalence patterns assuming there is no prior immunity to influenza in the swine population.

## Supplementary Material

Figure S3. Model-predicted persistence and seroprevalence patterns of swine influenza on finishing farms, allowing for infection of pigs with maternal antibodies.

## Supplementary Material

Figure S4. Sensitivity of model results to the number of sows and waning of maternal immunity on farrow-to-finish farms.

## Supplementary Material

Figure S5. Model-predicted persistence and seroprevalence patterns of swine influenza on combined site II/III finishing farms.

## Supplementary Material

Supplementary Data
